# Digital Education on Hospital Nutrition Diets: What Do Patients Want to Know?

**DOI:** 10.3390/nu16193314

**Published:** 2024-09-30

**Authors:** Neha Gutta, Som Singh, Dharti Patel, Aleena Jamal, Fawad Qureshi

**Affiliations:** 1 Department of Internal Medicine, University of Missouri Kansas City School of Medicine, Kansas City, MO 64108, USA; 2 Sidney Kimmel Medical College, Thomas Jefferson University, Philadelphia, PA 19107, USA; 3 Department of Nephrology and Hypertension, Mayo Clinic Alix School of Medicine, Rochester, MN 55905, USA

**Keywords:** hospital diets, patient education, nutrition, readability, digital education

## Abstract

Introduction: Therapeutic nutrition plays an imperative role during a patient’s hospital course. There is a tremendous body of literature that emphasizes the systematic delivery of information regarding hospital nutrition diets. A major component of delivering healthcare information is the principle of providing quality healthcare information, but this has not yet been investigated on hospital nutrition diets. This study aimed to evaluate the comprehension and readability of patient education materials regarding therapeutic hospital diets. Methodology: The methodology employed the use of publicly available questions regarding hospital nutrition diets and categorized them per Rothwell’s Classification of Questions. Additionally, the questions were extracted online and have an associated digital article linked to the question. These articles underwent analysis for readability scores. Results: This study’s findings reveal that most hospital diets do not meet the recommended grade-reading levels. Conclusions: This underscores the need for healthcare providers to enhance patient education regarding hospital diets. The prevalence of “Fact” questions showcases the importance of clearly explaining diets and dietary restrictions to patients.

## 1. Introduction

When a patient is admitted to an inpatient unit in the hospital, a critical component of their management plan is the evaluation and decision of providing an appropriate nutrition plan with regard to their clinical status diet plan. Therapeutic nutrition can help improve patients’ medical conditions or reduce risks associated with progression of their underlying medical condition [1]. Likewise, diets that are deemed not adequate or poor for individuals are associated with worse health outcomes [2,3]. From a clinical standpoint, there has been increased interest in developing feeding plans for patients that can aid during inpatient nutritional plans as well as serve as a vital pillar in preventative diseases as well as outpatient management [4,5,6,7].

Despite such a critical role in patient healthcare, the research funding within nutrition science remains lacking compared to other clinical disciplines [8,9]. From a public health sector, resource availability in the setting of food insecurity also plays a critical role in patient outcomes [7,10,11]. For clinicians, increased attention has been made towards innovative, medically tailored dietary plans for patients that contribute to improving their overall health [12]. However, the personalization of a diet plan becomes irrelevant if a patient is unable to understand their diet, which can cause a secondary lack of compliance with the diet as well [13]. In an effort to improve this understanding, clinicians must be able to evaluate the current tools important in aiding a patient in understanding their diet. One of these tools includes the use of digital modalities, such as the internet. The ever-rising ease and availability of internet-based tools provides patients with convenient, streamline sources of information on topics in patient education [14,15,16,17]. However, there is limited literature on the assessment of online patient educational tools regarding therapeutic nutrition diets, and there is a lack of regulation regarding the quality of these materials. This study aims to evaluate the quality of digital patient educational material on hospital nutrition diets.

## 2. Materials and Methods

The quality outcome of interest employed in the cross-sectional study was readability. Therefore, the aim of this study was to evaluate the readability of online, digital patient education materials regarding therapeutic hospital diets. Institutional review board approval was not necessary since neither human participants nor animal subjects were involved. Additionally, all the data that were used in this study were publicly available. RankBrain (Google, Mountain View, CA, USA), which is a machine learning-based search engine algorithm, contributed to this study as a means to find and extract the questions and associated digital educational materials extracted in this study [15,18,19,20,21]. Specifically, we queried the frequently asked questions and extracted the first unique 20 from the following categories similar to prior literature [20,22,23]: clear liquid diet, full-liquid diet, low-fat diet, low-salt diet, low-protein diet, consistent carbohydrate diet, mechanical soft diet, parenteral nutrition, soft diet, enteral nutrition, pureed diet, renal diet, and calorie-controlled diet. These categories were chosen based on common diet plans utilized in the inpatient setting in hospitals [24,25].

For questions and articles to be appropriately included in this study, the study authors evaluated each article to ensure that the article was written in English and that the article was accessible without needing to create a subscription account or to monetarily pay to view the article. Within the article, it must be at least 200 words long. These criteria were based on prior literature that tried to evaluate effective readability as well [21,26,27,28]. The 200-word minimum was supported based on McKenna et al., which reported that their readability calculations report a sample is appropriate when between 200 and 600 words [29]. After extracting the questions and their corresponding articles, two independent reviewers used Rothwell’s Classification of Questions, which categorizes the questions into either “Fact”, “Policy”, or “Value” based on the evaluation by the authors [30]. McCormick et al. outline descriptions of these classifications where a “Fact”-based question focused on evaluating the extent of a question as truth. “Policy”-based questions focused on evaluating a course of action, and “Value”-based questions focused on evaluating a concept [20,22,31]. Questions in the “Fact” category were subclassified as “restrictions”, “technical details”, “cost”, “timeline of recovery”, and “specific activities”. Questions in the “Policy” category were subclassified as “indications/management” or “risks/complications”. Questions in the “Value” category were subclassified as “evaluation”, “longevity”, and “evaluation of surgery” [30,32,33].

The associated articles were then all reformatted for standardization. Specifically, it was converted to plain text with 12-point font on Microsoft Word© (Version 16.89.1) as demonstrated in prior literature [21]. By standardizing the format, raters were able to calculate the readability scores from each article. Before calculating the readability scores and standard deviation (SD), author information, figures, captions, legends, references, copyright disclaimers, and website hyperlinks were removed [21]. Content was not revised or reviewed for source appraisal. Readability of the educational materials was assessed with the following readability scales: Coleman–Liau Index, Flesch Kincaid Grade-Reading Level, Flesch Reading Ease, Gunning–Fog Index Readability, Linsear Write Formula, and Simple Measure of Gobbledygook (SMOG) Index. These formulas were selected based on previous literature that has applied these scales across other applications in digital patient educational materials [34]. Moreover, the Flesch Kincaid Grade-Reading Level, Flesch Reading Ease, and Gunning–Fog have been applied to digital materials related to chronic medical conditions, including heart failure, procedural technology such as inferior vena cava filters, infections such as COVID-19, etc. [22,35,36]. In addition to these readability scales, the Coleman–Liau Index, Simple Measure of Gobbledygook (SMOG) Index, and Linsear Write Formula have been demonstrated in literature also regarding chronic diseases such as glaucoma, procedures such as bariatric surgery, etc. [37,38,39]. Given this depth of areas in patient education that these scales have been applied to, the decision was made to also apply them to nutritional diets as well. These readability scales are based on data points extracted from the text using their respective formulas. Frequent variables of interest in these formulas include the number of words, syllables, and sentences [34,40]. The Brief DISCERN instrument was also used on each educational resource to assess the quality of written information, and a cutoff of ≥16 was used [23,41,42]. By using these readability calculations along with the Brief DISCERN instrument, both the comprehension and quality of these educational materials for patients can be determined. Additionally, these calculations can help determine if the educational resource for each diet is at par with the United States grade-reading level recommendations of between a 6th-to-8th grade-reading level [43]. All data were recorded using Microsoft Excel 2021 (Microsoft Corporation, Redmond, WA, USA). To minimize potential ambiguity in data extraction, the date of the search queries was also tabulated [12,13,14].

## 3. Results

With the inclusion and exclusion criteria, there were 20 frequently asked questions and educational materials extracted for each of the 13 hospital diets studied, totaling 260 questions and educational materials used for this study. Of these materials, 89.2% (n = 232) were from the United States, 3.8% (n = 10) were from the United Kingdom, and the other countries included Ukraine, India, Australia, Canada, Scotland, and the Netherlands. Moreover, 38.5% (n = 100) of the educational materials came from commercial sources. Moreover, 21.2% (n = 55) of the materials came from medical practice, while 16.2% (n = 42) of the materials came from academic institutions. Media outlets contributed 14.2% (n = 37) of the materials. Government websites contributed the least to the materials extracted (10%, n = 26) (Table 1).

When Rothwell’s Classification of Questions was used, the majority of the frequently asked questions were classified as “Fact” (83.5%, n = 217). 8.8% (n = 23) of the questions were classified as “Value”, and a lower number of questions were classified as “Policy” (7.7%, n = 20). Upon further subclassification of the questions, 44.2% (n = 115) of all the frequently asked questions were about “Technical Details”, which is a subcategory of the “Fact” category. This was followed by “Restrictions”, which was also a subcategory of the “Fact” category and made up 34.2% (n = 89) of all the questions (Table 2). When assessing the readability of the educational materials, two articles were removed due to the inability to access the functional patient education articles. Six readability scores were calculated for 258 articles, resulting in a total of 1548 scores calculated. Educational materials on parenteral nutrition had the highest average Flesch–Kincaid score at 13.4 (SD = 3.8), while the materials on the renal diet had the lowest average Flesch–Kincaid score of 9.2 (SD = 1.5). When calculating the Flesch Reading Ease scores, the pureed diet has the highest average score of 63.1 (SD = 13), and parenteral nutrition has the lowest average score of 34.9 (SD = 20.1). Per the grade–reading level recommendation, the Flesch Reading Ease score should be ≥60 [15]. Moreover, 23.1% (n = 3) of the hospital diets that were studied met these recommendations. The hospital diets that had a Flesch Reading Ease score ≥ 60 included low-fat diet, soft diet, and pureed diet. Additionally, the brief DISCERN scores were found to be statistically significantly associated with Flesh Reading Ease when the scores met grade-reading level recommendations (*p* = 0.04) (Table 3).

Parenteral nutrition had the highest average of 16.2 (SD = 3.5) for the Gunning Fog score, and the full-liquid diet had the lowest average of 11.4 (SD = 2.4) for the Gunning Fog score. In regard to the Coleman–Liau Index, the highest average index score was 13.2 (SD = 3.2) with parenteral nutrition. Pureed diet had the lowest average index score of 8.9 (SD = 1.7). Parenteral nutrition also had the highest SMOG average of 12.4 (SD = 2.9). Low-fat diet had the lowest SMOG average of 8.5 (SD = 1.4). Parenteral nutrition had the highest linear write average score of 14.8 (SD = 5.2), and the renal diet had the lowest average linear write score of 19.2 (SD = 2.4).

## 4. Discussion

With increased access and reliance on the internet as a source of information, it is essential to evaluate the materials that individuals commonly use when seeking information [44]. Additionally, depending on each individual’s reading comprehension level and the readability of the articles they are using, patients can develop a dynamic understanding of their hospital diet, which may vary greatly. Therefore, this study helped evaluate the readability of online materials that may be used when seeking information regarding hospital diet plans so that improvements and changes can be made to provide better patient education on hospital diets.

Since the majority (89.2%) of the educational materials that were evaluated were from the United States, this study is most relevant to patients who are seeking medical care in the United States. Additionally, it is essential to consider the cultural aspect of diets from various cultures [45]. Therefore, it is difficult to compare the educational materials on hospital diets in the United States to other countries that may have different cultures, so this study may not be as relevant for individuals seeking medical nutritional care outside of the United States [46,47].

When utilizing Rothwell’s Classification of Questions, the majority (83.5%) of the questions were associated with the “Fact” category, which suggests that most individuals are trying to find out more information about hospital diets rather than looking up the policies or the value of the hospital diets. With “Technical Details” and “Restrictions” being the highest subclassification of the questions, this may suggest that individuals are more interested in learning the specific details about each of the diets and learning more about what they are unable to eat or drink with the specific hospital diets, but this requires further studies using survey-based methodologies to further encapsulate what individuals are interested in regarding diets.

The average scores from the readability analysis suggest that most diet plans do not meet the recommended reading level. It is recommended that the reading level should be less than or equal to that of a sixth-to-eighth grade-reading level [27,48,49]. Parenteral nutrition has the lowest average Flesch Reading Ease score of 34.9 (SD = 20.1), which suggests that it is difficult to read and is at the reading level of a college student [21,50]. Additionally, the statistically significant finding of the Brief DISCERN score, a measurement that evaluates the quality of a text based on six questions by the Brief DISCERN instrument, and a cutoff score to consider an article to be written of “quality” if the total score was ≥16 out of 30 total possible points [41]. This present study suggests that the greater difficulty in readability scores may be associated with the quality of the text itself [41,42].

Based on our understanding, this is one of the earliest cross-sectional studies that uses Rothwell’s Classification of Questions and the readability of online patient education materials on hospital diets in the United States. A strength of this study was the utilization of the Google RankBrain algorithm [51]. This algorithm contains over 90% of the market share of internet search queries, which suggests that we evaluated the most utilized materials that patients across the United States used when seeking information on hospital diets [21,52]. To increase the generalizability of these findings, additional internet search query programs may be used as well. Additionally, with social media usage being on the rise as a source of information for patients, it may be beneficial to assess the information on hospital diets that is posted on social media [53].

Although there are many strengths to this study, there are also limitations. The tools that were used in this study were well-established and assessed the comprehension and health literacy aspects of the educational materials. However, the tools do not assess the actual content for accuracy or actionability [40,54,55]. It would be helpful if a tool was developed that could assess the accuracy of online educational materials as well so that the amount of misinformation in these materials could also be evaluated. Since these assessment tools have a certain level of subjectivity, there may have been potential overlap between the categories when the questions were evaluated. The validity would increase with an increase in similar studies. With this study being a cross-sectional study, it only addresses the commonly used educational materials at the time it was conducted and does not account for or address any changes that could have occurred since then.

## 5. Conclusions

The findings of this study indicate that the grade-reading level recommendations have not been met on most of the hospital diets that were studied. This study demonstrates a need for healthcare providers, including dieticians, clinicians, and educators, to work on improving patient educational materials on hospital diets. Future directions to possibly improve this text could be in the use of new technological applications such as artificial intelligence [56,57]. The high frequency of “Fact” questions that the RankBrain algorithm created may suggest the importance of describing the diets and dietary restrictions to the patients. The low frequency of value- or policy-based questions suggests that healthcare providers are doing a good job of educating patients on things such as indications, risks, and longevity of these hospital diets. The results of this study also encourage providers to improve the online patient education materials on hospital diets to make them more readable for patients.

## Figures and Tables

**Table 1:** Source classification of educational materials regarding hospital nutritional diets.

Nutritional Diet	Academic Institution	Commercial	Government Website	Media Outlet	Medical Practice
Clear Liquid Diet	3	7	1	0	9
Full-Liquid Diet	4	7	0	0	9
Low-Fat Diet	3	13	0	3	1
Low-Salt Diet	2	12	3	3	0
Low-Protein Diet	3	11	1	3	2
Consistent Carbohydrate Diet	3	12	1	3	1
Mechanical Soft Diet	3	6	0	4	7
Parenteral Nutrition	6	5	5	0	4
Soft Diet	4	4	0	3	9
Enteral Nutrition	4	4	7	1	4
Pureed Diet	5	3	0	9	3
Renal Diet	1	5	6	2	6
Calorie-Controlled Diet	1	11	2	6	0

**Table 2:** Rothwell’s Classification of Questions regarding hospital nutritional diets.

Nutritional Diet	Fact	Policy	Value
Clear Liquid Diet	17	0	3
Full-Liquid Diet	19	0	1
Low-Fat Diet	19	0	1
Low-Salt Diet	17	3	0
Low-Protein Diet	14	5	1
Consistent Carbohydrate Diet	14	3	3
Mechanical Soft Diet	20	0	0
Parenteral Nutrition	13	5	2
Soft Diet	20	0	0
Enteral Nutrition	13	4	3
Pureed Diet	20	0	0
Renal Diet	19	0	1
Calorie-Controlled Diet	12	0	8

**Table 3:** Mean readability scores and standard deviations (SDs) regarding hospital nutritional diets.

Nutritional Diet	Flesch–Kincaid	Flesch Reading Ease	Gunning–Fog	Coleman–Liau Index	SMOG	Linsear Write
	Average	Average	Average	Average	Average	Average
	SD	SD	SD	SD	SD	SD
Clear Liquid Diet	9.9	58.5	11.8	9.3	9.1	11.8
	2.2	7.1	2.5	1.4	1.2	3.8
Full-Liquid Diet	9.4	59.6	11.4	9.7	8.8	10.9
	2.1	7.3	2.4	1.5	1.2	3.4
Low-Fat Diet	9.5	61.2	11.8	9.5	8.5	11.5
	2.5	9.9	2.6	1.4	1.4	4
Low-Salt Diet	10.7	57.1	13.4	9.5	9.8	13.3
	3.3	7.5	3.4	1.4	1.7	5.7
Low-Protein Diet	10.4	54.6	12.5	10.2	9.3	11.7
	2.2	9.4	2.3	1.2	1.5	3.6
Consistent Carbohydrate Diet	10.7	56.8	13.2	9.8	9.5	13.4
	2.8	12.1	2.5	2.8	1.8	4.2
Mechanical Soft Diet	10.9	58.3	13.2	10.2	9.3	14.1
	3.1	10.9	3.2	1.4	2	4.9
Parenteral Nutrition	13.4	34.9	16.2	13.2	12.4	14.8
	3.8	20.1	3.5	2.9	2.9	5.2
Soft Diet	10.1	60.9	12.2	10.1	8.8	12.9
	3.1	11.8	3.1	1.4	2.1	4.7
Enteral Nutrition	11.8	41.9	15	12.4	11	12.2
	2.7	15	2.8	2.3	2.1	4.2
Pureed Diet	9.6	63.1	11.7	8.9	8.3	12.1
	4.1	13	4.1	1.7	2.3	6.6
Renal Diet	9.2	58.9	11.8	9.9	8.7	10.2
	1.5	8.5	1.6	1.8	1.2	2.4

## Data Availability

The original contributions presented in the study are included in the article, further inquiries can be directed to the corresponding author.
